# Magnetic Nanoparticle Sensors

**DOI:** 10.3390/s91008130

**Published:** 2009-10-16

**Authors:** Isaac Koh, Lee Josephson

**Affiliations:** 1 T2 Biosystems, 286 Cardinal Medieros Ave, Cambridge, MA 02141, USA; E-Mail: ikoh@T2biosystems.com; 2 Center for Translational Nuclear Medicine, Department of Nuclear Medicine and Molecular Imaging and Center for Molecular Imaging Research, Massachusetts General Hospital/Harvard Medical School, 149 13th Street, Charlestown, MA 02129, USA

**Keywords:** magnetic particles, magnetic nanoparticles, target molecules, biosensors, magnetization

## Abstract

Many types of biosensors employ magnetic nanoparticles (diameter = 5–300 nm) or magnetic particles (diameter = 300–5,000 nm) which have been surface functionalized to recognize specific molecular targets. Here we cover three types of biosensors that employ different biosensing principles, magnetic materials, and instrumentation. The first type consists of magnetic relaxation switch assay-sensors, which are based on the effects magnetic particles exert on water proton relaxation rates. The second type consists of magnetic particle relaxation sensors, which determine the relaxation of the magnetic moment within the magnetic particle. The third type is magnetoresistive sensors, which detect the presence of magnetic particles on the surface of electronic devices that are sensitive to changes in magnetic fields on their surface. Recent improvements in the design of magnetic nanoparticles (and magnetic particles), together with improvements in instrumentation, suggest that magnetic material-based biosensors may become widely used in the future.

## 1. Introduction

Nanoscale magnetic materials are an important source of labels for biosensing due to their strong magnetic properties which are not found in biological systems. Modulation of the composition, size and magnetic properties of these materials permits their use in a variety of instruments and formats for biosensing [1,2]. New types of instrumentation are promising for the use of nanoscale magnetic materials in point of care sensors in variety of applications. Here, we cover three biosensors that employ magnetic nanoparticle labels with different sensing principles and instrumentation: (i) magnetic relaxation switches, (ii) magnetic particle relaxation sensors, and (iii) magnetoresistive sensors.

## 2. Magnetic Relaxation Switches (MRSws)

Superparamagnetic nanoparticles made of iron oxide and a polymeric coating are clinically proven magnetic resonance (MR) contrast agents and widely used in pre-clinical, targeted molecular imaging applications [2,3]. When used as targeted contrast agents, surface-modified nanoparticles (NPs) bind specific molecules producing local inhomogenieties in the applied magnetic field in tissues where molecular targets are present. These inhomogeneities result in decreases in the T_2_ relaxation time (or increases in 1/T_2_, the T_2_ relaxation rate), and these, in turn, lead to changes in the contrast of MR images.

**Figure 1.**
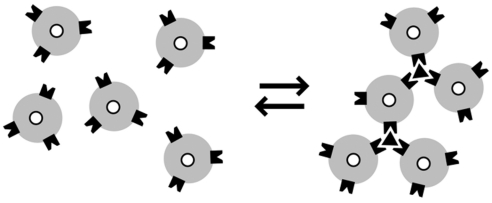
Principle of Type I MRSws. Dispersed magnetic nanoparticles (NPs) form an aggregate upon binding with target analytes (triangle). The aggregated form of the NPs dephases the spins of the surrounding protons of water molecules more efficiently than NPs present as the dispersed state. The effect is observed as a decrease in spin-spin relaxation time, T_2_ (reproduced with permission from reference [52]).

Recently, Josephson and collaborators exploited the change in T_2_ produced by magnetic NPs to obtain MR based assays called Magnetic Relaxation Switches (MRSws). The principle of MRSw assays is illustrated in Figure 1; NPs switch between dispersed and aggregated states, and associated with the change in aggregation are changes in the spin-spin relaxation time (T_2_), the basis of which is discussed below [4]. The materials used in MRSw assays are either magnetic nanoparticles (NPs, diameter 5–300 nm) or micometer-sized magnetic particles (MPs, diameter 300–5,000 nm). As shown in Figure 1, MRSws are homogeneous particle aggregation/disaggregation-based assays similar to aggregation assays using Latex particles, red blood cell hemagglutination, and antibody reactions with proteins (nephelometry). Unlike optically-based assays, MRSws employ radiofrequency radiation which penetrates biological samples regardless of their optical properties [5]. Since the dispersed and aggregated states of NPs (or MPs) can be reversed by such factors such as temperature, pH, and a high concentration of competing analytes, and hence are referred to as “relaxation switches”. The aggregated and dispersed states of magnetic NPs or MPs have different transverse spin-spin relaxation times (values of T_2_). NP aggregation and the size range of the resulting aggregates depends on the type of analyte and analyte concentration [6].

### 2.1. Mechanism of MRSws

MRSw assays exploit the fact that for both nanoparticles (NPs) and larger magnetic particles (MPs) transverse relaxation times (T_2_) differ between dispersed and aggregated states. However, for Type I, NP-based systems, T_2_ decreases with the aggregation, while with type II, MP-based systems T_2_ increases with aggregation. The basis of this is as follows [6–8].

The general theory of how magnetic spheres alter T_2_ is termed outer sphere relaxation theory. This theory uses two parameters of D_w_ and t_D_. D_w_ is the difference in angular frequencies between the local field experienced by a proton at the equatorial line of the sphere's surface and in the bulk (D_w_ = m_O_Mg/3, where m_O_ is the vacuum magnetic permeability, M is the particle magnetization, and g is the proton gyromagnetic ratio). Then t_D_ is the translational diffusion time of water around the sphere (t_D_ = R_a_^2^/D, where R_a_ is the sphere radius and D is the water diffusion coefficient). The outer sphere diffusion theory is applied when the motional average condition is fulfilled as D_w_t_D_ < 1 [7,8]. In this condition, the relaxation rate R_2_ (= 1/T_2_) increases as the sphere's size is increased. As the definitions of D_w_ and t_D_ imply, the motional average condition is not fulfilled with increased size of the particles such as MPs (D_w_t_D_ > 1) and the relaxation rate of 1/T_2_ decreases with the formation of MP aggregates. See the detailed discussion of this phenomenon in a review [8].

Thus, when present in solution magnetic NPs (or MPs) induce local magnetic field inhomogeneities, which cause a dephasing (loss of phase coherence) of the proton spin precession, and these inhomgeoneities lead to a reduction of the T_2_ relaxation time. When NPs aggregate (Type I MRSw), a smaller number of larger magnetic field inhomogeneities result. These larger inhomogeneities are more effective dephasers of proton relaxation and T_2_ drops. Here D_w_t_D_ < 1. When MPs aggregate (Type II MRSw), a smaller number of larger magnetic field inhomogeneities again results. However, there now so few aggregates, and spaces between them so great, that many water proteins fail to diffuse in and out of these homogeneities during the time course of the measurement. This is termed the “diffusion limited case” for the enhancement of proton relaxation by magnetic microspheres. Here D_w_t_D_ > 1.

Relaxivity is an important measure of the potency of magnetic materials and an important factor to selecting evaluating materials for use in MRSw assays. Materials with higher relaxivities are more detectable by the relaxometry and can detect lower concentrations of analyte [8].

(1) $${\text{R}}_{2}=(1/{\text{T}}_{2(+)}-1/{\text{T}}_{2(-)})/\text{C}$$

where R_2_ is relaxivity of the particle (in moles of metal) expressed as (mM sec)^−1^, C is the concentration of the paramagnetic center in mM, and 1/T_2(+)_ and 1/T_2(−)_ are the transverse relaxation rates (sec^−1^) in the presence and absence of the nanoparticle, respectively. C is typically expressed as the concentration of paramagnetic metal, but it can also be expressed as the concentration of NPs or MPs in solution. Here the R_2_ per metal is multiplied times the number of paramagnetic metal atoms per particle. Magnetic particles with larger numbers of metals per particle are more potent in MRSw assays, see below.

### 2.2. Magnetic Particles

Magnetic particles can be categorized by their size, with nanoparticles (NPs) being between 10 and 300 nm in diameter, while larger magnetic particles (MPs) are between 300 and 5,000 nm in diameter. Since the first publication demonstrating the MRSw assay principle in 2001 [4], NPs with surfaces of cross-linked iron oxide (CLIO) have been used for sensing for analytes ranging from small molecules to mammalian cells [5,9–12]. CLIO is an excellent NP both for *in vivo* MR imaging [13] and for MRSw assay applications, because of its stability in a variety of fluids, including aqueous buffers and blood, and because of its functional handle of amino groups. CLIO is prepared by two-step treatment of the monocrystalline iron oxide nanoparticle known as MION. The MION NP features a dextran coating which is first cross-linked with epichlorohydrin and then reacted with ammonia to obtain amino groups on the crosslinked dextran surface. MION and CLIO NPs have an iron oxide cores of about 5 nm in diameter and dextran shell (or crosslinked dextran shell) about 10 nm in thickness, so that both NPs have overall diameters between 25 nm and 30 nm.

**Figure 2.**
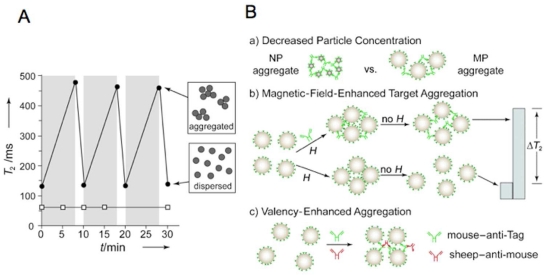
Methods for the improvement in MRSw assay sensitivities. (A) MPs (●) aggregate in a homogeneous magnetic field, whereas NPs (□) do not respond. A T_2_ increase in time is observed in a 0.47 T field (gray) in an MP solution, but not in an NP solution. The T_2_ value of the MP solution decreases as the MPs are dispersed with the field turn-off (white). Note that a T_2_ increase is observed with MP aggregation. (type II MRSw). Since this effect is slowed by the viscosity of the medium, T_2_-based viscometer can be obtained, see [7]. (B) Three strategies for enhancing the sensitivities with a type II MRSw assay. (a) A decreased concentration of MPs formed aggregates at a lower concentration of analyte (anti-Tag antibody) than that of NPs. MPs are larger than NPs and used at a lower concentration. (b) Application of a magnetic field (0.47 T) induced aggregation of MPs as in (A) and accelerated the interaction between MPs and analytes. (c) Target valency enhancement by addition of a secondary antibody (sheep anti-mouse). The valency increase of targets from two (anti Tag) to four (anti Tag:anti mouse) enhanced MRSw sensitivities. Figure reproduced with permission from reference [6].

Recently, magnetic NPs and MPs with improved magnetic properties, and higher detectability per particle, have been described for use with *in vivo* MR imaging and *in vitro* biosensor applications [1,14,15]. One strategy is to increase the R_2_ relaxivity of NPs by increasing M or d, since R_2_ is proportional to M^2^d^2^. Here M is the saturation magnetization per mole of metal or per gram of metal atoms within the particle and d is the particle diameter. [16–18]. Core/shell NPs have been designed with Fe metal cores (not iron oxide cores) and these have an increased Ms and a thin iron oxide shell to block oxidation metal oxidation. They show an enhanced sensitivity compared to CLIO for the detection of bacterial cells [17]. Another strategy employs Mn-doped metal oxide NPs; these also have high Ms and high R_2_s, and have been synthesized with sizes of 10, 12 and 16 nm. These NPs have been used in the sensitive detection of unprocessed cancer cells, with as few as two cells per 1 μL being detected with miniaturized relaxometer [16]. Another approach to improving the sensitivity of MRSw assays is the use of MPs rather than NPs. These MPs have far more metal atoms per particle than NPs and a far larger per magnetic moments per particle, even though their values or M per metal are typical of older NPs [6,19]. In an MRSw assay of immunoreactive antibodies to influenza, MPs of 1 μm in diameter were employed that had a similar R_2_ relaxivity to CLIO NPs on a per iron atom basis. However, the larger MPs had 350,000 fold more irons per particle than CLIO NPs. In the MRSw assay for anti-Tag peptide antibody, MPs had 186,000 fold enhanced sensitivity (relative to CLIO). The improvement in sensitivity was achieved by a combination of factors including the use of the larger MP, magnetic field-assisted aggregation of MPs, and valency enhancement achieved by the addition of a secondary antibody [19]. Figure 2B provides a schematic version of the improvement in assay sensitivity shows these techniques.

The stability of NPs or MPs in solution is another important factor in selecting materials for use in MRSw assay applications. Stabilization can be achieved by charge effects leading to electrostatic repulsion between particles or by the use of hydrophilic polymeric coatings that block particle/particle aggregation [8]. Coatings of polymeric dextran make NPs extremely stable and therefore suitable for both *in vivo* MR and *in vitro* MRSw assay applications [20]. Attachment of 10 kDa polyethylene glycol (PEG) diamine on the surface of MPs exchanged the initial electrostatic stability of the negatively charged MPs to polymer-based stability and was necessary to use the MPs in MRSw applications [19]. Table 1 reviews the magnetic particles used in MRSw biosensing applications.

### 2.3. Instrumentation

Point of care (POC) sensors would benefit home users, clinicians and physicians, and aid in the preparations for bio-warfare and pandemics. The miniaturization of MR relaxometers holds great promise for use as instrumentation with POC [10,16,17,21].

The MR relaxometers used for MRSw assays have three basic components, a magnet, a coil, and a transceiver. Currently MRSw assays depend on the commercial bench top relaxometers such as the 0.47 T Minispec, 20 MHz instrument made by Bruker, Billerica, MA [5, 19]. High throughput MRSw assays have been demonstrated in 384-well plates through the use of a 1.5 T MR scanner [5,22,23]. However, the relaxometer and MR scanner above are impractical as POC sensors due to their high cost, which results principally from the large magnets employed and lack of miniaturized electronic components [21].

**Figure 3.**
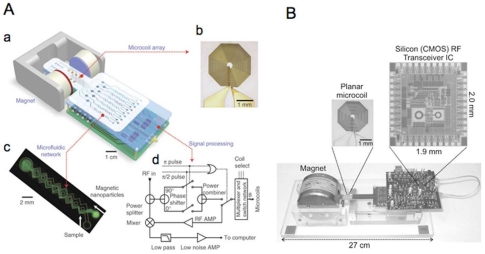
(A). Schematic representation of a miniaturized chip-based NMR system, diagnostic magnetic resonance (DMR). (B). NMR based CMOS RF biosensor. A complete NMR system was built with a portable platform (reproduced with permission (A) from reference [10] and (B) from reference [21]).

The magnets used in relaxometers can be relatively weak (0.1 to 0.5 T) and can provide less homogeneous magnetic fields than those used in MR imagers. One of the first miniaturized MR relaxometry systems consisted of a small palm-sized permanent magnet and on-board NMR electronics and planar microcoils with integrated microfluidic channels [10] (see Figure 3A). A multiplexed detection of biomarkers was achieved using an 8 microcoil array and demonstrated the potential application of the microNMR system for high throughput MRSw assays. Optimization of circuit designing in development of RF transceiver integrated circuits led to a small but complete NMR system [21] (Figure 3B).

### 2.4. Applications of Type I and Type II MRSw's

As discussed above, magnetic particle aggregation induces T_2_ changes that are opposite in direction for NPs (T_2_ decrease, type I MRSw) and MPs (T_2_ increase, type II, MRSw). Applications of the different types of MRSw systems are discussed below.

#### 2.4.1. Type I MRSw

The amino CLIO NP is a versatile NP for MRSw applications because it is sufficiently stable to permit a variety of surface chemistries [24–26]. CLIO surfaces have been designed to detect ions [12], DNA [4,5,27], proteins [5,9,19], and cells [10] such as bacteria and mammalian cells. A particularly valuable system for the study of MRSws is the reaction of NPs displaying the Tag peptide and reacting to a monoclonal antibody (anti Tag) binding to the peptide [6]. The formation of NP aggregates with anti-Tag antibodies has been shown to be analogous to the interactions between antibodies and antigens, with a maximum complex formation occurring at the equivalence point as the concentration of analyte was increased.

A variation of the type I MRSw aggregation/dispersion method is found with the miniaturized NMR system (DMR: diagnostic magnetic resonance). This system achieves a high assay sensitivity by reducing a sample volume to 5 μL and by using filtration methods [10]. Incorporation of microfluidic system with a filter unit into the miniaturized NMR system permitted the detection of bacteria, with as few as 20 colony-forming units per mL of sputum being detected [17]. The size discrepancy between target bacteria and NP probes allowed filter-based concentration of NP-bound bacteria while filtering out unbound NPs.

Another important application of the type I MRSw assay system is its use in an implantable MR based, water relaxation sensor. A semi-permeable membrane was employed with a size cutoff that permitted small analytes, like glucose, to diffuse in and out while the larger CLIO NPs were retained within the sensor [25]. Continuous monitoring of the T_2_ values of the solution inside the membrane showed a competitive assay type-response of glucose-functionalized CLIO to glucose [23,25]. The proof of concept sensing obtained with glucose was translated to an implantable water relaxation sensor detecting hCG as a cancer biomarker [28,29]. The implantable device had a reservoir that was covered with a semi-permeable polycarbonate membrane and contained CLIO functionalized with antibodies to the hCG cancer biomarker. *In vivo* MR imaging was used to monitor the T_2_ values from inside the sensor device. When implanted in a tumor bearing mouse model, the MR signal from the sensor showed significant decreases in 1-4 days due to diffusion of the cancer biomarker hCG into the reservoir and the resulting aggregation of the CLIO NP.

#### 2.4.2. Type II MRSw

Type II MRSws, where biomolecules are attached to MPs and aggregated by reaction with molecular targets, exhibit an increased T_2_ when aggregated by reaction with a target analyte. With their greater numbers of iron atoms per particle, MPs can be used at concentrations far below than that of NPs in MRSw assays. With the lower concentration of MPs, lower concentrations of analyte are needed to induce aggregation and this results in greatly improved sensitivity [6,19].

When placed in a homogeneous magnetic field, MPs with charge-based or polymer layer-based stability, will aggregate, while NPs will not respond in this fashion [6,7,19,30–33]. The magnetic field-induced MP aggregation is lost when the magnetic field was removed and Brownian effects break down aggregates. The rate of self-assembly formation of MPs in a magnetic field is a function of viscosity and can be used to make a T_2_ based viscometer. See Figure 2A and [7]. Recently, magnetic field-induced MP aggregation has also been used to accelerate analyte-mediated formation of MP aggregates [19,31,34]. The applied magnetic field enhanced the kinetics of molecular interactions between multivalent analytes, (e.g., a monoclonal Tag antibody), and multivalent MPs displaying the Tag peptide. This technique is referred to as magnetic field enhanced target aggregation and shown in Figure 2B, frame (b).

## 3. Magnetic Particle Relaxation-Based Sensors

The relaxation of the magnetic moments within magnetic particles have been used as a basis for magnetic particle-based assays.

### 3.1. Theory

Magnetic particles in a liquid, with magnetic moments aligned by an applied magnetic field, employ two relaxation mechanisms when magnetic field is turned off: (i) Brownian relaxation and (ii) Néel relaxation. Brownian relaxation is governed by the physical rotation of the entire particle and characterized by the Brownian relaxation time, τ_B_. Here:

(2) $$\tau_{\text{B}}=3{\text{V}}_{\text{H}}\eta /\text{kT}$$

where V_H_ is the hydrodynamic volume, η is the viscosity of the medium, k is the Boltzmann's constant, and T is the absolute temperature. The monodomain magnetic particle has an anisotropy energy, E_a_, which is proportional to the crystal volume.

(3) $${\text{E}}_{\text{a}}={\text{K}}_{\text{a}}\text{V}$$

where K_a_ is the anisotropy constant and V is the volume of the crystal. When the applied field is removed, the magnetization vector within the particle returns to the lowest energy state along the easy axis with a characteristic Néel relaxation time, τ_N_:

(4) $$\tau_{\text{N}}=\tau_{0}\exp({\text{E}}_{\text{a}}/\text{kT})$$

where τ_0_ is the preexponential factor that decreases as the anisotropy energy increases. Note that τ_N_ is an exponential function of the anisotropy energy that is proportional to the crystal volume.

The effective relaxation rate is expressed as the sum of the Brownian relaxation rate and the Néel relaxation rate:

(5) $$1/\tau =1/\tau_{\text{B}}+1/\tau_{\text{N}}$$

As Equation (5) shows, faster relaxation time between the two governs the effective relaxation process. Target induced aggregation can decrease the rates of the Brownian or Neel relaxations and this assays for molecular targets are generated. See [8].

### 3.2. Assays

#### 3.2.1. Néel Relaxation Sensors

**Figure 4.**
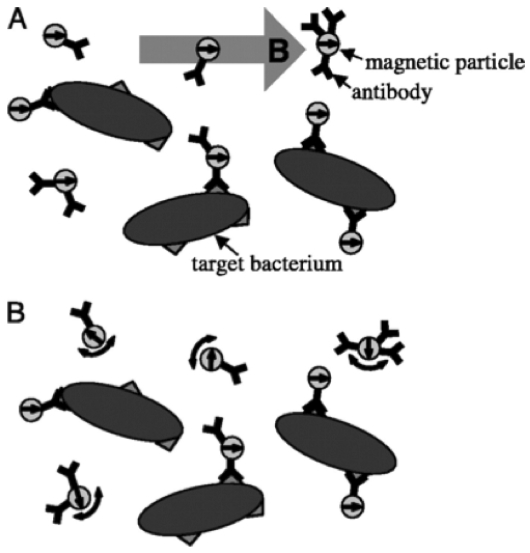
Principle of a SQUID-based homogeneous detector of bacteria. A. A pulse-form magnetic field orients the magnetic moments of NPs. B. After the field pulse is over, Brownian motion randomizes the magnetic moments of unbound NPs. However, the Brownian rotations of NPs bound to the bacteria are restricted. The bound NPs undergo Néel relaxation for reorientation of the magnetic moments. The SQUID detects the slower Néel relaxation for the bound NPs (reproduced with permission from reference [36]).

Superconducting Quantum Interference Devices (SQUIDs) have been used for measurements of the relaxation of particle magnetic moments. The Brownian relaxation is much faster than the Néel relaxation. For a 20 nm single domain magnetite particle in solution, the calculated relaxation times were τ_B_ ∼1 μs and τ_N_ ∼1 s [35]. The difference in the relaxation time scales was a basis for a homogeneous immunoassay [35] and a bacterial detection [36]. See Figure 4. The Brownian relaxation time scale of a single unbound magnetic particle was so short, it was out of the detectable range between 1 ms and 1 s of the SQUID. The free Brownian rotation of particles was then restricted when the magnetic particles bound a bacterium. The Néel relaxation was within the detection window of a SQUID, which was used to determine the relaxation time of surface bound particles. The SQUID-based detection of the Néel relaxation time showed a limit of detection of 5 × 10^4^ NPs for a substrate based assay and 1.1 × 10^5^ bacteria in a 20 μL sample volume. Development of a gradiometer instead of a magnetometer suggested a two-order improvement in sensitivity was possible [37].

#### 3.2.2. Brownian Relaxation Sensors

Measurements of static and dynamic magnetic susceptibility using alternate currents (ac) have permitted use of the Brownian relaxation of NPs for biosensing. As Equation (2) suggests, the NP aggregates that form in recognition of target analytes have a larger hydrodynamic size and thus show slower Brownian relaxation responses than a single NP. The resulting decrease in relaxation was sensed in buffer [38], and in serum [39] by using a SQUID or an ac magnetosusceptometer [39].

Conolly and St. Pierre proposed using the dynamic magnetic properties of NPs in assays [40]. A complex magnetic susceptibility expresses the response of NPs as a function of an alternating magnetic field. According to their theory, the imaginary part has a peak when the frequency equals the inverse of the effective magnetic relaxation time [41]. Brownian relaxation is the dominant relaxation process for these NP based assays. When NPs form molecular target induced aggregates, the hydrodynamic radius is increased and thus decrease of the peak frequency is observed. An antibody was detected at the sensitivity of 0.05 μg/mL (= 0.3 nM) with an AC susceptometer [42]. Recently a volume amplified magnetic nanobead assay showed a dynamic magnetic property-based detection of DNA detection, albeit with an amplification strategy [43]. Here the presence of two different size NPs in a sample provided a high detection of target DNA molecules following a deconvolution of the magnetization data [44].

## 4. Magnetoresistive Sensors

Magnetoresistive sensors are based on the binding of magnetic particles to a sensor surface and the magnetic fields of the particles alter the magnetic fields of the sensor which result in electrical current changes within the sensor. There are two mechanisms through which magnetic particles bind to the sensor surface: (i) direct labeling and (ii) indirect labeling (a sandwich type binding). Magnetic probes bind to the surface functionality on the surface in direct labeling by using streptavidin-biotin interaction or complementary DNA sequence recognition. Indirect labeling uses the principle of sandwich immunoassay in ELISA. For example, antibodies that bind to the target protein are immobilized on the surface. After treatment of the surface with a sample solution containing the target proteins, second antibodies that are biotinylated are added to the system. Finally Streptavidin coated magnetic particles are applied for tagging the biotinylated antibodies.

Giant magnetoresistance (GMR) spin valve (SV) or magnetic tunnel junction (MTJ) sensors have been successfully used to sense MPs. Sensors are composed of multiple layers of ferromagnetic materials. A biologically active molecule can be deposited on an Au layer or SiO_2_ layer to obtain a surface for the attachment of biomolecules. For a review of the structure of magnetoresistive sensors see [45].

**Figure 5.**
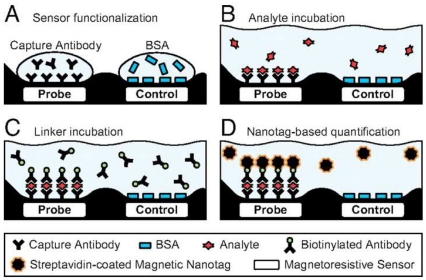
A schematic representation of a giant magnetoresistive (GMR) sensor for an ELISA-type protein assay. A. The probe surface was functionalized with a specific antibody, while the control surface was passivated with BSA. B. A sample solution was added for a specific binding of analyte proteins to the probe surface. C. A biotinylated antibody bound to the surface-immobilized analytes. D. Finally streptavidin-coated NPs were added for tagging the probe surface by biotin-streptavidin interaction. GMR signals were detected for sensing the presence of analytes on the surface. Courtesy from [48].

Superparamagnetic particles with different sizes have been used in magnetoresistive biosensing. Earlier applications used relatively large magnetic particles, with diameters between 0.1 and 3 μm [46]. Micrometer sized particles have the advantages of facile observation under light microscope and a higher particle-based magnetic moment that permits detection very small numbers of particles. However, recently magnetic NPs have replaced the larger particles because the NPs are stable in suspension and are less prone to particle clustering in an applied magnetic field [45,47–49]. Streptavidin coated MPs were applied to spin valve sensors in the protein marker detection at 27 pg/mL level of sensitivity [50]. By using 50 nm MACS magnetic nanoparticles, Wang and collaborators demonstrated cancer marker detection in 50% serum at sub picomolar concentrations [48] (Figure 5).

Improvement of spin valve sensors was achieved by reducing the passivation layer to 30 nm and led to an enhanced sensitivity. A signal amplification strategy that had multiple layers of streptavidin coated NPs and biotinylated antibodies in the sandwich type immunoassay also showed enhanced signals. Multiplex sensing of different protein markers in serum was demonstrated on a single chip by carefully selecting antibodies and by employing the signal enhancing strategy with multiple layers of NPs. Wang and his group in Standford University used nanoimprint lithography to synthesize antiferromagnetic nanoparticles of 100 nm size with high magnetic moment and zero remanence [51]. The antiferromagnetic nanoparticles that have a disk shape were composed of multiple layers of ferromagnetic material separated by a nonmagnetic interlayer. NPs with high magnetic moments were functionalized with streptavidin and permitted the detection of DNA at concentrations as low as 10 pM [47].

Another effort to synthesize magnetic nanoparticles with high magnetic moment utilized cubic-shaped FeCo nanoparticles of 12.8 nm in a GMR based sensor [49]. The cubic nanoparticles were surface functionalized with silane chemistry for attachment of Streptavidin or antibody. Direct labeling of biotinylated surface with Streptavidin coated nanoparticles allowed detection of 600 nanoparticle binding. Indirect labeling in ELISA type assay produced signals as low as 2 × 10^6^ molecules of a biomarker protein.

See Table 2 for a review of assay configurations and the sensitivities reported for them in the literature.

## 5. Conclusions

Magnetic NPs and MPs have been used in different types of biosensors based on different physical principles. Some achieve high sensitivity and, with rapid advances in instrumentation, maybe useful as point-of-care sensors. The continued rapid development of sensors using magnetic materials seems assured.

## Figures and Tables

**Table 1.** Characteristics of magnetic particles used for biosensing applications.

**Particle**	**Size**	**Composition**	**Characteristics**	**Reference**

CLIO	∼30 nm	5 nm core, 10 nm dextran coating	MRSw, R_2_ = 50 (s·mM Fe)^-1^	[5]
Core/shell	16 nm	Fe core, iron oxide shell, 2.5 nm shell thickness	MRSw, R_2_ = 260 (s·mM Fe)^-1^	[17]
Mn-MNP^a^	16 nm	Mn-doped iron oxide	MRSw, R_2_ = 420 (s·mM metal)^-1^	[16]
MP	1000 nm	Commercial (Dynabeads)	MRSw, R_2_ = 43 (s·mM Fe)^-1^	[19]
Iron oxide	56 nm	Commercial (Quantum Magnetics, Miltenyi Biotech)	SQUID	[35,36]
Iron oxide	19.5 nm		AC susceptometer	[42]
Cubic FeCo	12.8 nm	1.5 nm oxidized shell	GMR	[49]
SAF^b^	100 nm	Multilayers of ferromagnetic, interlayer of nonmagnetic material	GMR, disk shape	[47]
Magnetic bead	130, 250 nm	Commercial (Micromod Partikeltechnologie)	SQUID	[43]

(a) MNP: magnetic nanoparticle,

(b) SAF: synthetic antiferromagnetic.

**Table 2.** Sensitivities of magnetic particle based biosensors.

	**Analyte**	**Magnetic particle/instrumentation**	**Sensitivity**	**Sample volume**	**Reference**
MRSw type I	nucleotide	CLIO, bench top relaxometer	Low nM∼pM	300 μL	[4,5]
	proteins	CLIO, bench top relaxometer	Low nM	300 μL	[5,9]
	virus	CLIO, MRI	50 viruses/100 μL	100 μL	[11]
	bacteria	core/shell, DMR^a^	20 CFU^b^/100 μL (membrane filetered)	5 μL	[17]
	Cancer cell	Mn-MNP, DMR	2 cells/1 μL	5 μL	[16]
MRSw Type II	antibody	MP, bench top relaxometer	<1 pM	300 μL	[19]
AC susceptometer	antibody	Iron oxide NP	<1 nM		[42]
SQUID	bacteria	Iron oxide NP	1.1 × 10^5^ bacteria/20 μL		[36]
	DNA	Magnetic bead	3∼10 pM (signal amplification)		[43]
GMR	Protein	Cubic FeCo NP	2 × 10^6^ proteins	2 μL	[49]
	DNA	Antiferromagnetic NP	10 pM		[47]
	Protein	Iron oxide NP	2.4 pM		[48]

(a) DMR: diagnostic magnetic resonance,

(b) CFU: colony forming unit.
